# Simultaneous Determination and Stability Analysis of Ten New Psychoactive Substances including Synthetic Cathinones, Phenethylamines, and Ketamine Substitutes in Urine Using Liquid Chromatography-Tandem Mass Spectrometry

**DOI:** 10.1155/2023/9895595

**Published:** 2023-07-17

**Authors:** Feng-Shuo Yang, Hei-Hwa Lee, Li-Ping Tseng, Yung-Hung Lee, Yung-Sheng Lan, Yi-Cheng Lee, Yi-Cheng Chou, Yi-Ching Lin

**Affiliations:** ^1^Department of Laboratory Medicine, Kaohsiung Medical University Hospital, Kaohsiung Medical University, Kaohsiung 807, Taiwan; ^2^Department of Medicinal and Applied Chemistry, Kaohsiung Medical University, Kaohsiung 807, Taiwan; ^3^Department of Laboratory Medicine, School of Medicine, College of Medicine, Kaohsiung Medical University, Kaohsiung 807, Taiwan; ^4^Doctoral Degree Program of Toxicology, College of Pharmacy, Kaohsiung Medical University, Kaohsiung 807, Taiwan; ^5^Department of Medical Research, Kaohsiung Medical University Hospital, Kaohsiung Medical University, Kaohsiung 807, Taiwan

## Abstract

Knowing the stability of drugs is important to ensure accurate and reliable results of drug concentrations. This study evaluated the stability of ten new psychoactive substances (NPSs) in urine and methanol/water at different storage temperatures. Quantitative analyses were performed using liquid chromatography-tandem mass spectrometry. Three replicates of each storage condition were analyzed at day 0 and after 7, 14-, 30-, 60-, and 90 days with storage at +25°C, +4°C, and −20°C. For each analyte, the percent difference at each time interval from day 0 was calculated for each storage condition. Para-methoxyamphetamine (PMA), para-methoxymethamphetamine (PMMA), deschloroketamine (DCK), and 2-fluorodeschloroketamine (2-FDCK) were stable in urine, even when stored for 90-day periods at various temperatures. For synthetic cathinones, the concentrations declined over time at room temperature (+25°C) in urine but were relatively stable in methanol solvent with 0.1% formic acid. The significant degradation was found at +25°C, and the most excellent stability was shown by samples stored at −20°C. Phenethylamines (PMA and PMMA) and ketamine substitutes (DCK and 2-FDCK) were relatively more stable than synthetic cathinones (mephedrone, butylone, pentylone, ephylone, 4-MEAPP, and eutylone).

## 1. Introduction

New psychoactive substances (NPSs) are a complex and diverse group of substances often known as either designer or synthetic drugs or “legal highs.” NPSs are associated with several health and social harms on an individual and societal level [1, 2]. The number of NPSs is constantly growing, and trends and patterns of use change over time. Abuse of NPSs has become a critical threat to health and security in recent decades [3]. Some NPS, such as synthetic cathinones, para-methoxymethamphetamine, and ketamine, have posed a risk to public health and a challenge to drug policy [4]. Chewing khat leaves is common in the Arabian Peninsula and Eastern Africa. The primary psychoactive compositions in khat leaves cause sympathomimetic activity, mild euphoria, and excitation [5]. Synthetic cathinones are designer analogs of the natural active principle of khat. In recent years, synthetic cathinones have become the most seized class of NPS reported to the United Nations Office on Drugs and Crime (UNODC) Early Warning Advisory System [6]. Para-methoxymethamphetamine (PMMA) and para-methoxyamphetamine (PMA) are the para-methoxylated analogs of methamphetamine and amphetamine and have been found in tablets and capsules of the 3,4-methylenedioxymethamphetamine (MDMA) sold as “ecstasy.” A number of deaths have been attributed to tablets that contained PMMA and PMA [7–9]. 2-fluorodeschloroketamine (2-FDCK) and deschloroketamine (DCK) as substitutes for ketamine have emerged among drug abusers in recent years and are considered to have similar abuse potential as ketamine [10]. Due to its recent appearance, little research has been done on the compound.

The urine specimen is commonly used for NPS analysis. The time from sample collection to analysis might be several days due to transportation time. Sometimes, repeat testing due to unexpected results or additional drug analysis makes the time between collection and analysis increase by more than several days. Because of this time interval, to ensure that results are accurate, it is necessary to demonstrate that specimens are stable during this time frame [11, 12]. Moreover, the preparation of quality control and calibration samples in the process of illicit drug analysis is time-consuming. The working solutions of the analyte reference materials were often prepared using methanol and water and then stored for several days to months at refrigerator or freezer temperature. If preanalytical changes in concentration occur between specimen collection and analysis or between the initial and retests due to drug instability or degradation, the results of the re-test may vary from the original test results [13, 14]. Therefore, understanding the stability of analyzed drugs in testing samples is critically important.

Most published studies on NPS stability focused on single-category [14–18]. Few studies addressed the stability among different NPS categories. The stability studies among different solvents were also scarce. Methods to detect NPSs in biological matrices are challenging due to their low concentrations. Liquid chromatography-tandem mass spectrometry (LC-MS/MS) has become a tool of choice for quantitative bioanalytical assays due to its inherent selectivity and sensitivity and has proven to be fast and accurate [19, 20]. In this study, we used LC-MS/MS to develop a quantitative method for simultaneous determination of multiple NPSs and evaluate the stability of these NPSs, classified as synthetic cathinones, phenethylamines, and ketamine, in 50% methanol/water with or without 0.1% formic acid, and urine, at different storage temperatures.

## 2. Materials and Methods

### 2.1. Chemicals and Reagents

Reference materials were used for the validation and stability analysis. All chemicals and solvents used were of analytical grade. Methanol was purchased from Fisher Chemicals (Loures, Portugal), formic acid was purchased from Merck (Darmstadt, Germany), and ethanol was purchased from Merck (Darmstadt, Germany). 4-methylmethcathinone (mephedrone), butylone, pentylone, eutylone, ephylone, 4-methyl-*α*-ethylaminopentiophenone (4-MEAPP), 4-methoxyamphetamine (PMA), para-methoxymethamphetamine (PMMA), deschloroketamine (DCK), mephedrone-D3, butylone-D3, 3,4-methylenedioxyamphetamine-D5 (MDA-D5), and ketamine-D4 were purchased from Cerilliant (Texas, USA). 2-fluorodechloroketamine (2-FDCK) was purchased from Cayman Chemicals (Ann Arbor, MI, USA).

### 2.2. Sample Preparation

The study was conducted according to the guidelines of the Declaration of Helsinki and approved by the Institutional Review Boards of Kaohsiung Medical University Hospital (KMUHIRB-E(I)-20221077). After informed consent was obtained, the midstream urine was obtained from five healthy volunteers simultaneously and pooled together for validation and stability experiments. Therefore, the urine matrices in this study were the same in all test samples. Pooled blank urine was confirmed to be negative for any medications using LC-MS/MS testing and stored at −20°C. The calibration standards and quality control samples were prepared using pooled blank urine. The working solution of ten analytes (10 mg/L) was prepared in 50% methanol/water with 0.1% formic acid from reference materials of each analyte. Spiking solutions were prepared from the working solutions as mixtures of the ten NPSs. The internal standards (ISs), including mephedrone-D3, butylone-D3, MDA-D5, and ketamine-D4, were prepared in 50% methanol/water with 0.1% formic acid for each final concentration with 25 ng/mL.

Protein precipitation was performed before LC-MS/MS analysis. In a microcentrifuge tube, 50 *μ*L of urine was mixed with 50 *μ*L methanol. After centrifugation at 14,000 × *g* for 10 min to separate layers, 20 *μ*L of the supernatant was transferred to a new microcentrifuge tube and mixed with 160 *μ*L of 25 ng/mL internal standard and 20 *μ*L of 50% methanol/water and 0.1% formic acid solution. Then, 20 *μ*L of the sample was injected into the LC-MS/MS system.

### 2.3. Liquid Chromatography-Tandem Mass Spectrometry (LC-MS/MS)

Chromatographic separation was performed using an ACQUITY® UPLC® H-Class UPLC system (Waters, USA) with a Kinetex Biphenyl LC column (100 mm × 2.1 mm, 2.6 *μ*m, Phenomenex) preceded by a Security Guard™ ULTRA Cartridge UHPLC Biphenyl 2.1 mm ID column. The mobile phases comprised solvents A and B: 2% and 99.9% methanol in 0.1% formic acid solution, respectively. The gradient elution profile is shown in Table 1. The analytes were then quantified using a Xevo® TQ-XS tandem mass spectrometer (Waters Corporation, Milford, MA US). The mass spectrometer was operated in multiple reaction monitoring modes (MRMs). The MRM transitions and conditions for the analytes and ISs, as well as the retention times, target ions, and qualifier ions used for identification and quantification, are shown in Table 2.

### 2.4. Method Validation

The validation procedures were based on guidelines for bioanalytical method validation [21, 22] and the SWGTOX validation guidelines for urine [23]. All method validations were carried out by LC-MS/MS, and the data were analyzed by Microsoft Excel software (version 15.0.5363.1000).

#### 2.4.1. Specificity and Selectivity

Specificity and selectivity were assessed by spiking each drug and individual IS to test for interference. Exogenous interferences were evaluated by spiking blank matrices with twelve analytes, including dehydronorketamine, ethylone, dibutylone, flunitrazepam, nimetazepam, nitrazepam, phenylpropanolamine, ephedrine, pseudoephedrine, phentermine, cocaine, and ecgonine methyl ester, to ascertain if exogenous analytes may interfere with the method analytes.

#### 2.4.2. Sensitivity and Linearity

The lower limit of quantification (LLOQ) was determined as the lowest concentration with chromatographic signal-to-noise (S/N) value of 10 : 1 and met the accuracy limit of ±20%. Linearity was assessed by analyzing ten separate calibration curves by spiking blank urine with concentrations at 3.1, 6.2, 12.5, 25, 50, 62.5, 75, and 100 *μ*g/L for all compounds. Calibration curves produced per batch were generated by plotting the peak area ratio (PAR) versus the spiked analyte concentration. A blank matrix containing only IS was analyzed with each batch but not included in the calibration curves. The correlation coefficient (*R*^2^) was calculated and with an acceptability criteria of >0.99.

#### 2.4.3. Precision and Accuracy

Accuracy and precision were calculated by running calibration standards alongside five replicates of 25, 50, and 75 ng/mL. Both intra- and interday precision were assessed across five batches with an acceptability criterion of ≤10%. A method accuracy limit of ±15% was used [24].

#### 2.4.4. Carryover and Matrix Effects

Carryover was assessed by running the highest calibration standard (H) and analyzing subsequent blanks of 50% methanol/water with 0.1% formic acid (L) for the presence of any analytes in the sequence L1, L2, L3, H1, H2, L4, H3, H4, L5, L6, L7, L8, H5, H6, L9, H7, H8, L10, H9, H10, and L11. The difference between the high-low and low-low means needs to be less than three times the low-low SD.

According to the guideline on bioanalytical method validation [22], the matrix effects were estimated with ten urine samples from ten individuals. For each analyte and the IS, the matrix factor (MF) was calculated for each sample by calculating the ratio of the peak area in the presence of the matrix (measured by analyzing a matrix blank spiked after extraction with an analyte) to the peak area in the absence of the matrix (pure solution of the analyte). The IS-normalized MF was also calculated by dividing the analyte's MF by the IS's MF. The CV of the IS-normalized MF from the ten different urine samples should not be greater than 15%.

### 2.5. Sample Stability

Analyte stability was analyzed in different matrices and temperatures, including solutions of 50% methanol, 50% methanol/water with 0.1% formic acid, and urine, and storage at 25°C (room temperature), 4°C (refrigerator), and −20°C (freezer). Stability was assessed by monitoring the PARs of each analyte to the IS and the individual peak areas themselves. Three replicates at each concentration were analyzed at day 0 and after 7, 14, 30, 60, and 90 days. For each analyte, the percent difference at each time interval from day 0 was calculated and averaged for triplicate samples at each storage condition. There were no exclusion criteria; the percent difference calculations included every specimen concentration result. A concentration change of ±20% was deemed unstable.

## 3. Results and Discussion

### 3.1. Validation Experiments

#### 3.1.1. Specificity and Selectivity

**Figure 1:**
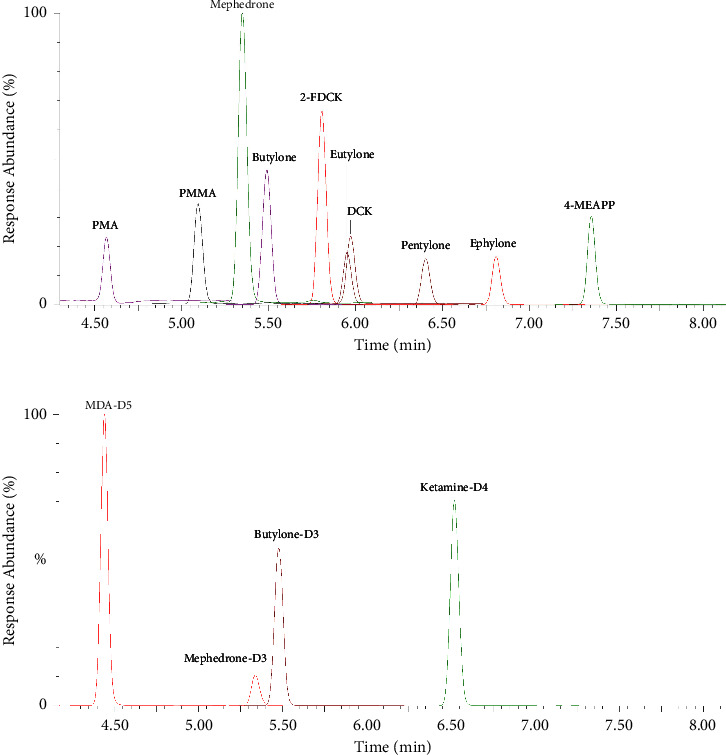
Total ion chromatogram of the analytes. The total ion chromatogram (TIC) of (a) 10 new psychoactive substances and (b) four internal standards in this study. Mephedrone: 4-methylmethcathinone; ephylone: N-ethylpentylone; 4-MEAPP: 4-methyl-*α*-ethylaminopentiophenone; PMA: para-methoxyamphetamine; PMMA: para-methoxymethamphetamine; DCK: deschloroketamine; 2-FDCK: 2-fluorodeschloroketamine; MDA-D5: 3,4-methylenedioxyamphetamine-D5.

The retention time, MRM transitions, dwell time, cone voltage, and collision energy for the analytes and ISs are shown in Table 2. The total ion chromatogram (TIC) of the analytes and internal standards are shown in Figure 1. No endogenous or exogenous interferences were observed from the pooled drug-free matrices analyzed. None of the analytes or ISs interfered with the other analytes' peak areas or retention times within this method.

#### 3.1.2. Sensitivity and Linearity

The LLOQs of all analytes in this method validation are shown in Table 3. 4-MEAPP, eutylone, and 2-FDCK were linear over a concentration range of 3.1–100 *μ*g/L with a correlation coefficient (*R*^2^) > 0.99. The calibration curves for other analytes were linear over a concentration range of 6.3–100 *μ*g/L (*R*^2^ > 0.99). The linearity curves of all analytes are shown in Figure S1.

#### 3.1.3. Precision and Accuracy

According to quantitative mass spectrometry for pharmacokinetic studies, the bioanalytical method of choice must be specific, precise, and reproducible to the intended analyte in a given matrix . The current best industry practice of validating an LC-MS/MS method and applying it for sample analysis suggested that accuracy is determined by replicate analysis of samples (QCs) containing known amounts of the analyte. The mean measured value should be within 15% (bias) of the nominal value for all QC concentration levels except for the LLOQ, where the bias (%) should be within 20% [24, 25].

The accuracy and precision results are shown in Table 3. All analytes in urine had precision values (%CV) <10%. The means of intraday precision (%CV) of all analytes in urine were 3.32, 2.58, and 2.85% at 25, 50, and 75 ng/mL, respectively. The interday precision across all analytes averaged 5.32%, 5.84%, and 4.76% at 25, 50, and 75 ng/mL, respectively. The accuracy of each analyte in urine fell within the ±15% criterion with a range from 96.20% to 108.80%.

#### 3.1.4. Carryover and Matrix Effects

No carryover was found in the blank samples following duplicate injection of the highest calibrator. The concentrations of all blank samples were 0 *μ*g/L. Matrix effect data of all analytes are shown in Table 4. The matrix effects of these analytes ranged from 84%–109%.

### 3.2. Stability of the Analytes in Different Matrices and Temperatures

**Figure 2:**
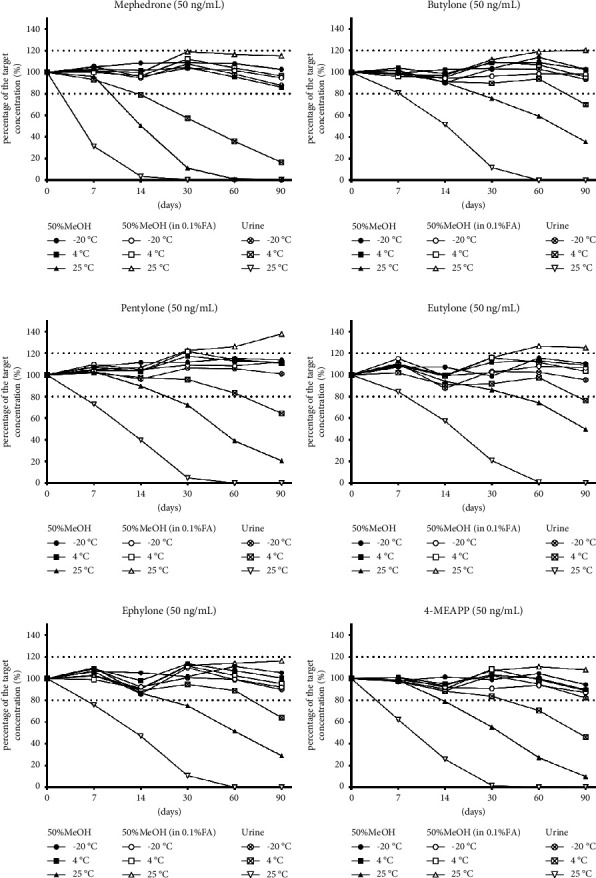
The stability of six synthetic cathinones in different matrices and temperatures. The stability of (a) 4-methylmethcathinone (mephedrone), (b) butylone, (c) pentylone, (d) eutylone, (e) N-ethylpentylone (ephylone), and (f) 4-methyl-*α*-ethylaminopentiophenone (4-MEAPP) in 50% methanol/water (50% MeOH), 50% methanol/water with 0.1% formic acid (50% MeOH in 0.1% FA), and urine at −20°C, 4°C, and 25°C.

**Figure 3:**
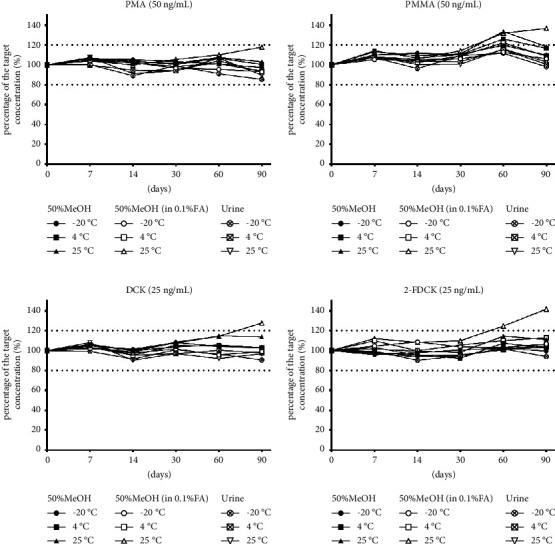
The stability of phenethylamines and ketamine substitutes in different matrices and temperatures. The stability of (a) para-methoxyamphetamine (PMA), (b) para-methoxymethamphetamine (PMMA), (c) deschloroketamine (DCK), and (d) 2-fluorodeschloroketamine (2-FDCK) in 50% methanol/water (50% MeOH), 50% methanol/water with 0.1% formic acid (50% MeOH in 0.1% FA), and urine at −20°C, 4°C, and 25°C.

The stability of each analyte in different matrices and temperatures are shown in Figures 2 and 3. The details of the percentage of the target concentration for the analytes in different matrices and storage temperatures are shown in Table S1.

#### 3.2.1. The Stability Study of Mephedrome

The stabilities of the synthetic cathinones in different matrices and storage temperatures are shown in Figure 2. For mephedrone, when the samples were stored at freezer temperature (−20°C), the percent differences were within 20% of the original measurement for all solvents, even by day 90. When the samples were stored in 50% methanol/water with 0.1% formic acid, the percent differences were within 20% of the original measurement for all storage temperatures for 90 days (Figure 2(a)). Mephedrone in urine stored at 25°C showed a 68.65 ± 1.21% loss of the initial concentration by day 7 and a 96.43 ± 0.06% loss of the initial concentration by day 14. Mephedrone in urine stored at 4°C showed a 20.9 ± 3.66% loss of the initial concentration by day 14 and a 42.66 ± 3.10% loss of the initial concentration by day 30 (Table S1).

#### 3.2.2. The Stability Study of Butylone, Pentylone, and Eutylone

For butylone, pentylone, and eutylone, when the samples were stored at −20°C, the percent differences were within 20% of the original measurement for all solvents, even by day 90. When stored in 50% methanol/water (with or without 0.1% formic acid), the percent differences were within 20% of the original measurements at 4°C and −20°C (Figures 2(b)–2(d)).

Butylone in urine stored at 25°C showed a 48.42 ± 1.90% loss of the initial concentration by day 14 and an 88.15 ± 0.73% loss of the initial concentration by day 30. Butylone in urine stored at 4°C showed a 30.01 ± 1.64% loss of the initial concentration by day 90. Butylone in 50% methanol/water without 0.1% formic acid stored at 25°C showed a 24.43 ± 1.61% loss, a 40.74 ± 1.03% loss, and a 64.50 ± 0.31% loss of the initial concentration by day 30, day 60, and day 90, respectively.

Pentylone in urine stored at 25°C showed a 27.22 ± 2.19% loss of their initial concentration by day 7 and a 60.16 ± 1.44% loss of the initial concentration by day 14. Pentylone in urine stored at 4°C showed a 35.54 ± 1.24% loss of the initial concentration by day 90. Pentylone in 50% methanol/water without 0.1% formic acid stored at 25°C showed a 27.84 ± 1.19% loss, a 61.03 ± 1.70% loss, and a 79.37 ± 0.69% loss of the initial concentration by day 30, day 60, and day 90, respectively.

Eutylone in urine stored at 25°C showed a 42.74 ± 3.10% loss of the initial concentration by day 14 and a 79.26 ± 1.09% loss of the initial concentration by day 30. Eutylone in urine stored at 4°C showed a 23.92 ± 0.64% loss of their initial concentration by day 90. Eutylone in 50% methanol/water without 0.1% formic acid stored at 25°C showed a 25.78 ± 2.29% loss, and a 50.21 ± 0.37% loss of the initial concentration by day 60, and day 90, respectively (Table S1).

#### 3.2.3. The Stability Study of Ephylone and 4-MEAPP

For ephylone and 4-MEAPP, when the samples were stored at −20°C, the percent differences were within 20% of the original measurement for all solvents, even by day 90. When the samples were stored in 50% methanol/water with 0.1% formic acid, the percent differences were within 20% of the original measurement for all storage temperatures for 90 days (Figures 2(e) and 2(f)).

Ephylone in urine stored at 25°C showed a 24.32 ± 0.90% loss of the initial concentration by day 7 and a 53.00 ± 1.85% loss of the initial concentration by day 14. Ephylone in urine stored at 4°C showed a 36.11 ± 1.47% loss of the initial concentration by day 90. Ephylone in 50% methanol/water stored at 25°C showed a 25.17 ± 2.29% loss of the initial concentration by day 30 and a 71.02 ± 0.41% loss of the initial concentration by day 90.

4-MEAPP in urine stored at 25°C showed a 37.81 ± 1.52% loss of the initial concentration by day 7 and a 74.29 ± 1.69% loss of the initial concentration by day 14. 4-MEAPP in urine stored at 4°C showed a 29.59 ± 1.28% loss of their initial concentration by day 60 and a 53.98 ± 1.09% loss of the initial concentration by day 90. 4-MEAPP in 50% methanol/water stored at 25°C showed a 21.05 ± 3.44% loss of the initial concentration by day 14 and a 90.3 ± 0.19% loss of the initial concentration by day 90 (Table S1).

#### 3.2.4. The Stability Study of PMA and PMMA

PMA and PMMA samples were stable for more than 90 days at 4°C and −20°C in all solvents evaluated in this study (Figures 3(a) and 3(b)). In PMA samples, the percent differences were all within 20% of the original measurements in all solvents, even at room temperature by day 90. In PMMA samples, a more than 20% difference was found only in urine at room temperature by day 60.

#### 3.2.5. The Stability Study of DCK and 2-FDCK

DCK and 2-FDCK samples were stable for more than 90 days at 4°C and −20°C in all solvents evaluated in this study (Figures 3(c) and 3(d)). In the DCK and 2-FDCK samples, more than 20% differences were found only in 50% methanol/water with 0.1% formic acid at room temperature by day 90 and day 60, respectively.

**Figure 4:**
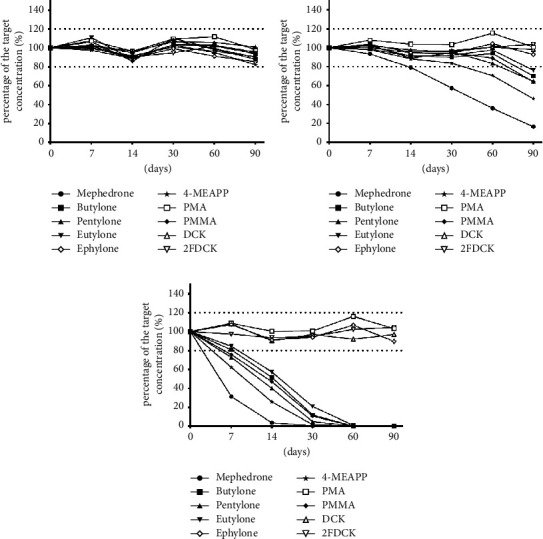
Comparison of the stability of the analytes in urine at different temperatures. Stability of the 10 analytes in urine at (a) frozen temperature (−20°C), (b) refrigerated temperature (4°C), and (c) room temperature (25°C). The targeted concentrations for mephedrone, butylone, pentylone, eutylone, ephylone, 4-MEAPP, PMA, and PMMA are 50 ng/mL. The targeted concentrations for DCK and 2-FDCK are 25 ng/mL. Mephedrone: 4-methylmethcathinone; ephylone: N-ethylpentylone; 4-MEAPP: 4-methyl-*α*-ethylaminopentiophenone; PMA: para-methoxyamphetamine; PMMA: para-methoxymethamphetamine; DCK: deschloroketamine; 2-FDCK: 2-fluorodeschloroketamine.

The stability study showed that phenethylamines (PMA and PMMA) and ketamine substitutes (DCK and 2-FDCK) were relatively stable than synthetic cathinones (mephedrone, butylone, pentylone, ephylone, 4-MEAPP, and eutylone). PMA, PMMA, DCK, and 2-FDCK were stable in urine, even when stored for 90-day periods at various temperatures (Figure 4). Similar results were reported previously about the long-term stability of amphetamine-type stimulants (ATS) [26, 27]. Thus, delayed testing, repeat testing, and add-on testing for PMA, PMMA, DCK, and 2-FDCK in urine specimens can yield reliable results for up to 90 days following the urine collection date.

The stability results for synthetic cathinones showed that the detectable concentrations would decline over time (Figure 2). In urine samples, all the synthetic cathinones tested in this study declined within 7–14 days at room temperature. Most synthetic cathinones, except mephedrone, are stable in urine for at least 30 days when stored at refrigerator temperature. Mephedrone concentrations in urine significantly declined within 14 days, even at refrigerator temperatures (Figure 4). All tested synthetic cathinones were stable at freezer temperature in both urine and 50% methanol/water, no matter with or without 0.1% formic acid. Synthetic cathinones dissolved in 50% methanol with 0.1% formic acid were relatively more stable than those stored without formic acid in the present study. Previous studies reported similar results that synthetic cathinones are relatively stable under acidic conditions [14].

Many commercially available NPS reference substances are dissolved in methanol. Considering the polarity and solubility distribution of NPSs, we chose 50% methanol as one of the solvents for drug stability evaluation. Some previous studies that investigated the stability of synthetic cathinones used preservatives or buffers containing metal ions for different pH solutions [14, 28]. However, metal ions may interfere with mass spectrometry or damage the instrument. In this study, we used formic acid to maintain the pH of solvents.

The chemical behavior of synthetic cathinone is mainly determined by the ketone and amine groups in its structure. Synthetic cathinones have a “beta-keto” structure and two tautomers of “keto” and “enol” form in solution. Many factors, such as solvent properties, matrix, pH value, aromaticity, conjugation, hydrogen bonds, and substitutions of synthetic cathinones, would affect the equilibrium position between the keto and enol forms [29]. A previous study considered that solvent effects are due to the strong tendency of the enol form to hydrogen bond intramolecularly, while the keto form may hydrogen bond with protic solvents, providing stabilization [30]. Acidic conditions favor the formation of an enol form. It has been reported that acid makes the carbonyl group more electrophilic, increasing the acidity of alpha-protons and facilitating enol formation [29]. Previous studies demonstrated that mephedrone could form dihydro-mephedrone through carbonyl reduction of its ketone group [31, 32]. We speculate that synthetic cathinones might partially be transformed into the enol form due to low pH in 50% methanol with 0.1% formic acid. The reaction of the ketone functional group will not be able to proceed under acidic conditions, thus leading to an increase in stability (Figure S2).

Using unstable reference material might lead to unreliable results that could significantly impact the justice system and the individuals whose samples are tested in casework. Storing the samples with 0.1% formic acid would improve the stability of the analytes, especially in synthetic cathinones. The most significant degradation was found at room temperature (25°C), and the most excellent stability was stored in the freezer (−20°C). Although unopened reference material may be stable for long, this work highlights the importance of regularly updating reference material once opened.

## 4. Conclusion

In this study, we simultaneously determined ten NPSs, including synthetic cathinones, phenethylamines, and ketamine substitutes, and evaluated their stability in various solvents and storage temperatures. Mephedrone is the least stable analyte tested in this study. Phenethylamine and ketamine substitutes are more stable than synthetic cathinones, synthetic cathinones are more stable under acidic conditions, and all analytes are stable within 90 days at freezer (−20°C) temperature. Knowing the stability of drugs is essential to ensure accurate and reliable results of drug concentrations. These findings highlight the importance of the storage environment for reference materials and biological samples in forensic laboratories when performing NPS analysis.

## Figures and Tables

**Table 1:** Time program for the chromatographic separation procedure.

Total time (min)	Flow rate (mL/min)	Mobile phase solvent A (%)	Mobile phase solvent B (%)
0	0.4	95	5
1	0.4	95	5
4	0.4	70	30
7	0.4	50	50
13.5	0.5	5	95
14	0.5	5	95
14.5	0.4	95	5
16	0.4	95	5

Solvent A: 97.9% water with 2% methanol and 0.1% formic acid. Solvent B: 99.9% methanol with 0.1% formic acid.

**Table 2:** Retention time, multiple reaction monitoring transitions, dwell time, cone voltage, collision energy for analytes, and internal standards.

Compound name	Retention time (min)	Precursor ion (m/z)	Product ion (m/z)	Dwell time (ms)	Cone voltage (V)	Collision energy (V)	Internal standard
Mephedrone	5.38	178.00	160.00	0.003	18	12	Mephedrone-D3
			145.00		18	22	

Butylone	5.54	222.10	174.10	0.003	25	15	Butylone-D3
			204.10		30	10	
Pentylone	6.47	236.00	188.00	0.008	24	20	Butylone-D3
			218.00		24	12	

Eutylone	6.01	236.00	188.00	0.008	36	25	Butylone-D3
			218.00		36	19	

Ephylone	6.89	250.00	202.00	0.008	31	26	Butylone-D3
			174.00		31	41	

4-MEAPP	7.43	220.00	105.00	0.022	12	26	Butylone-D3
			144.00		12	38	

PMA	4.60	166.00	149.00	0.003	25	8	MDA-D5
			121.00		25	20	

PMMA	5.15	180.10	120.91	0.003	30	22	MDA-D5
			91.10		30	30	

DCK	6.04	204.00	173.00	0.008	21	17	Ketamine-D4
			145.00		21	24	

2-FDCK	5.88	222.00	109.00	0.008	22	25	Ketamine-D4
			163.00		22	25	

Mephedrone-D3	5.37	180.79	147.89	0.003	40	18	—
			144.83		38	20	

Butylone-D3	5.53	225.03	177.07	0.003	36	18	—
			148.94		36	26	

MDA-D5	4.47	185.00	168.00	0.003	20	10	—
			110.00		20	20	

Ketamine-D4	6.59	242.00	129.00	0.008	20	30	—
			224.00		20	20	

LC-MS/MS: liquid chromatography-tandem mass spectrometry; mephedrone: 4-methylmethcathinone; ephylone: N-ethylpentylone; 4-MEAPP: 4-methyl-*α*-ethylaminopentiophenone; PMA: para-methoxyamphetamine; PMMA: para-methoxymethamphetamine; DCK: deschloroketamine; 2-FDCK: 2-fluorodeschloroketamine; MDA-D5: 3,4-methylenedioxyamphetamine-D5.

**Table 3:** Lower limit of quantification (LLOQ), calibration curve, correlation coefficient (*R*^2^), precision, and accuracy of all analytes in urine by LC-MS/MS.

Analytes	LLOQ (ng/mL)	Calibration curve (ng/mL)	*R * ^2^	Intraday precision (*n* = 5, %CV)			Interday precision (*n* = 5, %CV)			Accuracy (%) (*n* = 5)		
				25 ng/mL	50 ng/mL	75 ng/mL	25 ng/mL	50 ng/mL	75 ng/mL	25 ng/mL	50 ng/mL	75 ng/mL
Mephedrone	6.3	6.3–100.0	0.9969	5.66	3.85	3.08	2.23	4.16	2.31	100.00	101.20	108.80
Butylone	6.3	6.3–100.0	0.9991	1.80	3.27	4.37	6.63	1.90	5.81	99.20	100.40	97.80
Pentylone	6.3	6.3–100.0	0.9984	2.83	3.59	3.24	4.87	9.21	4.66	100.00	99.60	98.40
Eutylone	3.1	3.1–100.0	0.9986	4.90	2.30	3.09	4.83	3.03	5.20	100.00	96.20	98.40
Ephylone	6.3	6.3–100.0	0.9994	3.64	2.95	3.37	8.33	9.15	9.30	98.40	100.40	98.40
4-MEAPP	3.1	3.1–100.0	0.9991	3.37	2.83	3.75	4.53	7.35	4.04	99.20	100.00	97.33
PMA	6.3	6.3–100.0	0.9990	4.67	1.08	1.20	5.48	9.58	3.04	97.60	101.20	99.47
PMMA	6.3	6.3–100.0	0.9997	2.23	1.77	0.94	8.70	6.96	5.24	98.40	100.80	100.00
DCK	6.3	6.3–100.0	0.9997	0.89	0.90	2.00	3.52	3.04	4.34	101.60	99.96	98.93
2-FDCK	3.1	3.1–100.0	0.9994	2.83	2.60	2.87	2.23	4.66	3.56	100.00	101.87	98.67

LC-MS/MS: liquid chromatography-tandem mass spectrometry; mephedrone: 4-methylmethcathinone; ephylone: N-ethylpentylone; 4-MEAPP: 4-methyl-*α*-ethylaminopentiophenone; PMA: para-methoxyamphetamine; PMMA: para-methoxymethamphetamine; DCK: deschloroketamine; 2-FDCK: 2-fluorodeschloroketamine; CV: coefficient of variation.

**Table 4:** Matrix factor (MF) in % (range) of all analytes in urine.

Compound name	MF (50 ng/mL)		CV (%) of the IS-normalized MF^*∗*^
Mephedrone	94.8	[82–110]	8.6
Butylone	99.1	[92–109]	4.9
Pentylone	94.9	[90–104]	3.8
Eutylone	98.1	[86–109]	7.2
Ephylone	93.5	[86–101]	4.5
4-MEAPP	95.0	[90–104]	4.6
PMA	87.8	[82–103]	8.6
PMMA	95.5	[85–103]	5.5
DCK	93.3	[84–103]	5.1
2-FDCK	95.5	[91–99]	2.6

^*∗*^The CV(%) of the IS-normalized MF calculated from ten different samples of the matrix should not be greater than 15%. CV: coefficient of variation; IS: internal standard; mephedrone: 4-methylmethcathinone; ephylone: N-ethylpentylone; 4-MEAPP: 4-methyl-*α*-ethylaminopentiophenone; PMA: para-methoxyamphetamine; PMMA: para-methoxymethamphetamine; DCK: deschloroketamine; 2-FDCK: 2-fluorodeschloroketamine.

## Data Availability

The data presented in this study are available on request from the corresponding author. Data may be available upon request to interested researchers. Please send data requests to Yi-Ching Lin, MD, PhD, Department of Laboratory Medicine, Kaohsiung Medical University Hospital, Kaohsiung Medical University.

## Supplementary Materials

Supplementary Materials Figure S1: calibration curves for the analytes in this stury. All the calibration curves were assessed at 3.1, 6.2, 12.5, 25, 50, 62.5, 75, and 100 ng/mL. Figure S2: keto-enol tautomers and carbonyl reduction from the keto tautomer of mephedrone. Table S1: the details of the percentage of the target concentration for the analytes in different matrices and storage temperatures.
