# Supplementary material for: Simultaneous Determination and Stability Analysis of Ten New Psychoactive Substances including Synthetic Cathinones, Phenethylamines, and Ketamine Substitutes in Urine Using Liquid Chromatography-Tandem Mass Spectrometry
Source: Int J Anal Chem. 2023 Jul 17;2023:9895595. doi: 10.1155/2023/9895595 (PMC10365918; doi:10.1155/2023/9895595)
Supplement: Supplementary Materials — Figure S1: calibration curves for the analytes in this stury. All the calibration curves were assessed at 3.1, 6.2, 12.5, 25, 50, 62.5, 75, and 100 ng/mL. Figure S2: keto-enol tautomers and carbonyl reduction from the keto tautomer of mephedrone. Table S1: the details of the percentage of the target concentration for the analytes in different matrices and storage temperatures. [file 9895595.f1.pdf]

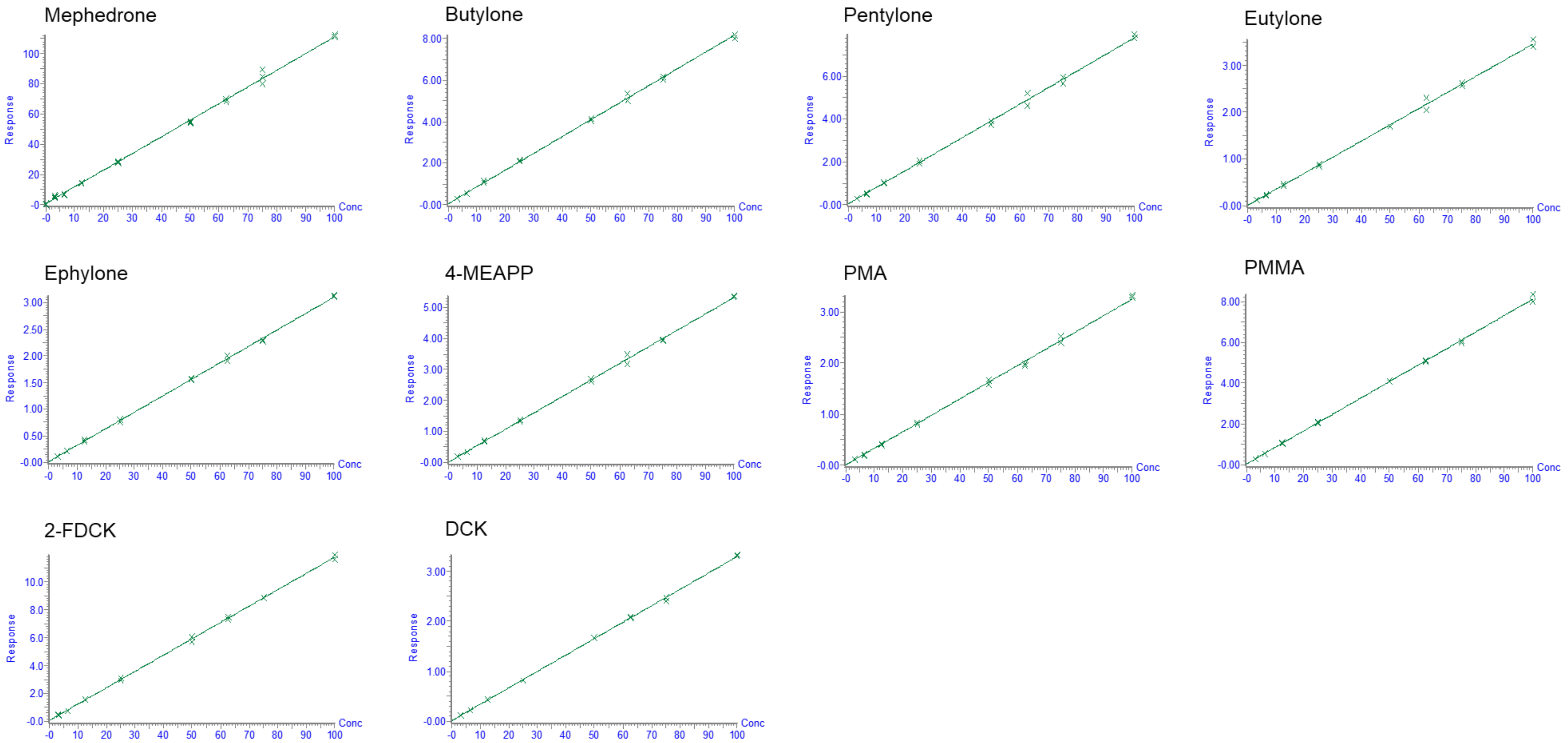

Figure S1. Calibration curves for the analytes. All the calibration curves were assessed at 3.1, 6.2, 12.5, 25, 50, 62.5, 75, and 100 ng/mL. Mephedrone: 4-methylmethcathinone; Ephylone: N-ethylpentylone; 4-MEAPP: 4-methyl-α-ethylaminopentiophenone; PMA: para-methoxyamphetamine; PMMA: para-methoxymethamphetamine; DCK: deschloroketamine; 2-FDCK: 2-fluorodeschloroketamine.

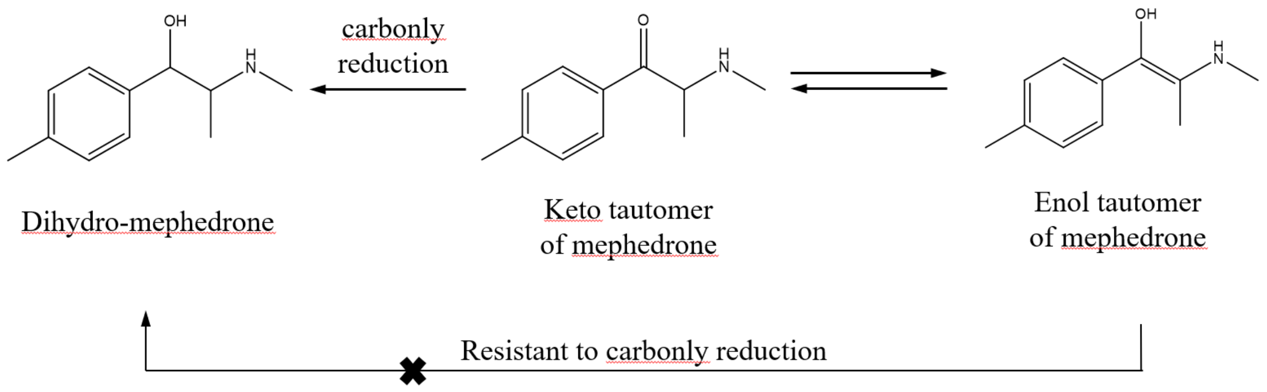

Figure S2. Keto-enol tautomers and carbonyl reduction from the keto tautomer of mephedrone.

Table S1: The details of the percentage of the target concentration for the analytes in different matrices and storage temperatures.

**Compound**	**Solvent**	**Storage temperature (℃)**	**% difference between days 0 and 7**	**% difference between days 0 and 14**	**% difference between days 0 and 30**	**% difference between days 0 and 60**	**% difference between days 0 and 90**	**First unstable day of analysis**
Mephedrone (50 ng/mL)	50%MeOH	-20	4.88±4.35	8.48±0.61	8.89±5.47	7.87±0.89	2.78±2.29	--
		4	3.26±3.08	1.70±1.50	4.41±10.3	-4.34±3.70	-13.84±5.62	--
		25	-3.93±2.67	**-49.53±1.62**	**-88.87±0.82**	**-98.91±0.12**	**-99.53±0.24**	day 14
	50%MeOH (in 0.1%FA)	-20	5.16±3.67	-3.98±3.07	3.98±2.26	1.70±5.57	-5.09±2.75	--
		4	0.00±6.18	-3.26±5.80	12.14±9.89	4.05±7.09	-3.33±1.00	--
		25	1.24±3.46	-0.07±3.76	19.06±2.04	16.51±1.04	15.01±6.15	--
	Urine	-20	1.98±4.24	-5.36±2.53	7.67±8.16	-1.65±6.49	-12.17±3.85	--
		4	-6.81±4.53	**-20.90±3.66**	**-42.66±3.10**	**-63.96±0.94**	**-83.60±0.03**	day 14
		25	**-68.65±1.21**	**-96.43±0.06**	**-99.67±0.30**	**-99.93±0.11**	**-99.60±0.20**	day 7
Butylone (50 ng/mL)	50%MeOH	-20	-0.87±1.01	2.55±1.21	3.49±3.39	13.76±5.28	2.75±2.56	--
		4	3.83±1.93	-1.34±3.69	8.59±3.85	8.86±6.96	2.55±7.67	--
		25	1.54±3.38	-9.46±1.28	**-24.43±1.61**	**-40.74±1.03**	**-64.5±0.31**	day 30
	50%MeOH (in 0.1%FA)	-20	-0.71±1.25	-5.63±1.54	-3.69±5.75	-1.49±4.60	-1.75±2.70	--
		4	-4.01±2.61	-4.72±6.99	9.64±11.38	6.86±8.11	-4.40±0.98	--
		25	-2.14±3.70	-2.98±2.79	11.65±1.08	18.90±4.28	**20.39±6.70**	day 90
	Urine	-20	1.48±2.96	-9.79±2.28	2.96±4.95	2.90±4.85	-6.63±1.10	--
		4	-1.67±5.99	-8.82±6.91	-10.11±2.71	-6.31±1.90	**-30.01±1.64**	day 90
		25	-19.25±4.42	**-48.42±1.90**	**-88.15±0.73**	**-100±0.00**	**-99.87±0.11**	day 14
Pentylone (50 ng/mL)	50%MeOH	-20	7.57±4.87	11.49±1.10	11.71±2.58	15.27±4.87	13.85±6.89	--
		4	7.07±4.42	3.43±1.83	17.42±8.15	12.92±5.07	11.56±7.15	--
		25	2.78±0.37	-10.35±2.79	**-27.84±1.19**	**-61.03±1.7**	**-79.37±0.69**	day 30
	50%MeOH (in 0.1%FA)	-20	9.62±6.87	4.85±1.82	9.05±3.15	8.34±5.43	12.19±3.33	--
		4	3.92±2.06	3.49±5.35	9.48±15.16	14.04±5.79	10.83±0.54	--
		25	4.92±2.79	6.91±4.53	**22.59±0.99**	**26.09±1.46***	**37.78±4.86**	day 30
	Urine	-20	2.73±1.88	-3.96±3.62	6.21±8.05	5.66±4.65	0.89±1.24	--
		4	1.77±5.76	-2.39±4.69	-4.30±6.42	-16.71±2.56	**-35.54±1.24**	day 90
		25	**-27.22±2.19**	**-60.16±1.44**	**-95.23±0.24**	**-100.00±0.00**	**-100.00±0.00**	day 7
Eutylone (50 ng/mL)	50%MeOH	-20	7.20±2.15	7.13±2.88	-1.20±2.67	15.68±2.04	10.45±6.36	--
		4	8.05±2.12	0.21±3.92	11.65±4.60	12.85±4.53	9.04±8.90	--
		25	8.97±5.02	-5.93±1.32	-13.70±3.35	**-25.78±2.29**	**-50.21±0.37**	day 60
	50%MeOH (in 0.1%FA)	-20	15.04±1.17	-0.85±0.77	2.27±6.37	7.94±4.02	6.52±7.35	--
		4	8.79±6.67	-1.21±6.11	15.89±10.61	11.56±6.86	3.26±1.60	--
		25	10.00±4.61	-0.43±2.22	16.03±7.89	**26.74±3.54**	**25.18±10.92**	day 60
	Urine	-20	10.67±5.32	-12.33±2.54	3.25±1.28	2.45±0.75	-4.64±1.69	--
		4	1.79±3.91	-9.28±5.27	-8.08±2.49	-2.52±3.38	**-23.92±0.64***	day 90
		25	-15.51±1.73	**-42.74±3.10**	**-79.26±1.09***	**-99.14±0.11**	**-99.80±0.00**	day 14
	50%MeOH	-20	6.46±2.58	5.17±2.32	1.77±0.66	11.09±1.71	5.17±2.86	--

		4	8.78±2.35	-1.77±2.93	13.47±2.96	7.01±5.61	0.61±6.65	--
		25	9.80±2.35	-13.54±2.18	**-25.17±2.29**	**-48.57±2.16**	**-71.02±0.41**	day 30
Ephylone (50 ng/mL)	50%MeOH (in 0.1%FA)	-20	8.76±3.82	-7.73±3.00	0.77±1.86	-0.71±5.42	-8.05±3.10	--
		4	-0.97±2.82	-9.47±6.23	11.34±5.76	2.64±5.13	-4.77±3.06	--
		25	2.90±3.70	-9.15±2.53	11.92±4.19	14.05±0.51	16.11±1.96	--
	Urine	-20	6.19±1.67	-14.15±0.52	9.91±6.38	-1.04±5.38	-10.17±2.36	--
		4	2.54±7.06	-11.08±2.93	-5.48±4.77	-11.08±1.37	**-36.11±1.47**	day 90
		25	**-24.32±0.90**	**-53.00±1.85**	**-89.44±0.52**	**-99.93±0.11**	**-99.93±0.11**	day 7
4-MEAPP (50 ng/mL)	50%MeOH	-20	-2.09±1.85	1.40±0.50	-1.08±5.49	4.50±2.58	-5.64±3.11	--
		4	0.89±1.81	-5.01±2.45	3.30±1.90	-0.06±3.88	-10.34±6.34	--
		25	-1.59±2.23	**-21.05±3.44**	**-44.71±1.05**	**-72.92±1.40**	**-90.3±0.19**	day 14
	50%MeOH (in 0.1%FA)	-20	0.86±3.10	-8.12±0.91	-9.23±5.65	-6.28±2.68	-13.05±1.51	--
		4	-2.22±5.00	-9.60±5.84	8.55±9.79	-0.98±6.76	-11.14±2.62	--
		25	-1.23±1.33	-6.34±4.06	7.2±1.57	10.71±4.06	7.94±1.02	--
	Urine	-20	-2.76±4.13	-11.41±1.42	2.82±5.97	-5.20±6.97	-17.87±5.16	--
		4	-2.26±4.80	-11.85±5.40	-16.36±2.14	**-29.59±1.28**	**-53.98±1.09**	day 60
		25	**-37.81±1.52**	**-74.29±1.69**	**-98.43±0.11**	**-100.00±0.00**	**-99.69±0.29**	day 7
PMA (50 ng/mL)	50%MeOH	-20	5.85±1.90	5.37±4.00	3.33±2.12	0.88±1.93	-2.65±1.87	--
		4	6.94±2.41	4.08±1.27	0.27±6.20	6.87±3.70	0.34±1.36	--
		25	6.67±4.17	2.93±1.74	1.50±2.26	7.55±2.32	3.13±3.06	--
	50%MeOH (in 0.1%FA)	-20	4.30±2.14	2.22±1.72	-3.36±0.35	-4.51±3.78	-7.06±3.04	--
		4	5.78±0.91	1.75±0.91	-1.41±3.79	1.14±1.51	-6.39±2.62	--
		25	4.10±2.25	-0.13±0.61	5.51±2.71	10.09±1.72	17.82±1.45	--
	Urine	-20	0.19±1.35	-11.05±1.56	-0.96±5.00	-9.06±1.24	-14.72±0.68	--
		4	-0.06±1.46	-5.46±0.59	-6.11±2.42	4.18±1.42	-7.39±3.32	--
		25	7.39±1.84	-8.87±3.41	-6.17±2.06	6.88±1.31	-10.48±0.87	--
PMMA (50 ng/mL)	50%MeOH	-20	10.13±1.56	11.69±1.12	10.35±1.79	19.73±1.69	9.75±1.49	--
		4	14.15±2.63	6.92±1.52	10.35±5.28	12.58±0.59	16.60±3.97	--
		25	13.33±1.86	9.23±3.88	11.02±0.77	11.69±2.68	18.32±3.35	--
	50%MeOH (in 0.1%FA)	-20	5.44±1.76	5.08±1.21	6.09±4.04	12.69±0.58	6.09±2.63	--
		4	7.47±0.78	2.47±2.30	9.14±4.26	14.94±0.33	8.92±3.91	--
		25	7.76±2.90	6.02±3.33	14.14±8.92	**31.91±3.18**	**36.84±1.32**	day 60
	Urine	-20	7.36±4.61	-3.79±1.46	9.36±3.94	11.72±4.34	-2.00±0.43	--
		4	8.01±2.44	3.65±1.26	3.29±3.90	15.30±4.41	0.64±1.38	--
		25	8.72±3.54	0.29±1.73	0.57±3.33	16.01±0.43	3.00±3.84	--
DCK (25 ng/mL)	50%MeOH	-20	4.74±5.28	1.35±1.24	8.12±0.85	4.19±1.64	3.25±4.30	--
		4	6.09±4.71	-0.41±1.17	3.79±2.70	5.55±0.81	2.71±1.46	--
		25	6.22±2.94	0.27±0.00	7.71±1.88	14.61±3.10	13.80±1.54	--
	50%MeOH (in 0.1%FA)	-20	2.14±2.85	-2.27±3.24	1.60±4.10	-4.41±1.90	-0.94±3.83	--
		4	6.15±1.52	-4.68±2.28	5.21±1.41	4.28±1.45	2.81±4.10	--
		25	3.07±4.19	-1.47±3.24	8.56±2.35	14.30±1.45	**27.67±6.22**	day 90
	Urine	-20	-0.79±2.05	-8.66±3.88	0.92±4.14	-3.67±4.60	-9.58±3.88	--
		4	3.28±5.84	-4.72±0.79	-3.15±4.17	0.26±3.55	-1.97±1.97	--
		25	8.14±5.59	-9.84±1.42	-3.02±1.27	-8.14±0.60	-3.15±3.88	--

2-FDCK (25 ng/mL)	50%MeOH	-20	-4.15±5.00	-2.07±2.16	0.52±2.92	2.33±0.22	-0.52±5.64	--
		4	-1.94±4.47	-4.79±3.75	-7.77±1.84	7.38±5.25	2.72±4.28	--
		25	1.55±1.57	-0.65±2.37	-1.68±3.11	14.51±2.50	11.14±6.25	--
	50%MeOH (in 0.1%FA)	-20	4.52±4.87	8.76±4.00	3.67±10.59	3.11±4.33	6.64±6.97	--
		4	9.89±3.40	-0.14±0.65	5.51±1.53	10.03±3.60	12.99±1.29	--
		25	12.15±3.60	8.19±3.29	9.89±8.29	**24.58±0.85**	**41.67±2.82**	day 60
	Urine	-20	-0.66±5.84	-9.84±2.58	-5.38±1.86	1.71±5.52	-6.04±2.17	--
		4	3.02±6.05	-4.59±3.30	-4.46±1.49	0.66±4.70	3.54±1.72	--
		25	-2.76±4.77	-6.43±0.82	-5.25±4.34	2.23±2.77	4.59±4.60	--

Bold* values indicate the change in concentration > 20%. Mephedrone: 4-methylmethcathinone; Ephylone: N-ethylpentylone; 4-MEAPP: 4-methyl-α-ethylaminopentiophenone; PMA: para-methoxyamphetamine; PMMA: para-methoxymethamphetamine; DCK: deschloroketamine; 2-FDCK: 2-fluorodeschloroketamine; 50%MeOH: 50% methanol and 50% water; 50%MeOH (in 0.1%FA): 50% methanol/water with 0.1% formic acid.
